# Caffeic Acid Phenethyl Ester and Its Amide Analogue Are Potent Inhibitors of Leukotriene Biosynthesis in Human Polymorphonuclear Leukocytes

**DOI:** 10.1371/journal.pone.0031833

**Published:** 2012-02-09

**Authors:** Luc H. Boudreau, Jacques Maillet, Luc M. LeBlanc, Jacques Jean-François, Mohamed Touaibia, Nicolas Flamand, Marc E. Surette

**Affiliations:** 1 Département de chimie et biochimie, Université de Moncton, Moncton, Canada; 2 Centre de recherche de l'institut universitaire de cardiologie et de pneumologie de Québec (IUCPQ), Département de médecine, Faculté de médecine, Université Laval, Québec, Canada; University of Leuven, Rega Institute, Belgium

## Abstract

**Background:**

5-lipoxygenase (5-LO) catalyses the transformation of arachidonic acid (AA) into leukotrienes (LTs), which are important lipid mediators of inflammation. LTs have been directly implicated in inflammatory diseases like asthma, atherosclerosis and rheumatoid arthritis; therefore inhibition of LT biosynthesis is a strategy for the treatment of these chronic diseases.

**Methodology/Principal Findings:**

Analogues of caffeic acid, including the naturally-occurring caffeic acid phenethyl ester (CAPE), were synthesized and evaluated for their capacity to inhibit 5-LO and LTs biosynthesis in human polymorphonuclear leukocytes (PMNL) and whole blood. Anti-free radical and anti-oxidant activities of the compounds were also measured. Caffeic acid did not inhibit 5-LO activity or LT biosynthesis at concentrations up to 10 µM. CAPE inhibited 5-LO activity (IC_50_ 0.13 µM, 95% CI 0.08–0.23 µM) more effectively than the clinically-approved 5-LO inhibitor zileuton (IC_50_ 3.5 µM, 95% CI 2.3–5.4 µM). CAPE was also more effective than zileuton for the inhibition of LT biosynthesis in PMNL but the compounds were equipotent in whole blood. The activity of the amide analogue of CAPE was similar to that of zileuton. Inhibition of LT biosynthesis by CAPE was the result of the inhibition of 5-LO and of AA release. Caffeic acid, CAPE and its amide analog were free radical scavengers and antioxidants with IC_50_ values in the low µM range; however, the phenethyl moiety of CAPE was required for effective inhibition of 5-LO and LT biosynthesis.

**Conclusions:**

CAPE is a potent LT biosynthesis inhibitor that blocks 5-LO activity and AA release. The CAPE structure can be used as a framework for the rational design of stable and potent inhibitors of LT biosynthesis.

## Introduction

5-lipoxygenase (5-LO), expressed in a number of myeloid and lymphoid cells such as B cells, monocytes, neutrophils, eosinophils and mast cells, is the key enzyme in the bioconversion of arachidonic acid (AA) to leukotrienes (LTs) [1]. LTs are important lipid mediators of inflammation that are involved in various inflammatory diseases such as atherosclerosis [2], asthma [3] and rheumatoid arthritis [4]. Studies have also demonstrated a potential role for 5-LO in cancer since its overexpression is observed in tissue samples from patients with prostate carcinoma [5] and this enzyme is an important regulator of leukemia stem cell development [6]. Consequently, the inhibition of the 5-LO pathway has been studied as a therapeutic target for a number of years (reviewed by [7]). The anti-asthmatic drug zileuton [8] is the only 5-LO inhibitor approved and commercially available for clinical use, but adverse effects including liver toxicity has limited its use [9]. Another inconvenience of the drug is its pharmacokinetic profile requiring dosing of up to 600 mg four times a day [8], [10]. Thus the search for alternative and potent 5-LO inhibitors with fewer side effects continues.

**Figure 1:**
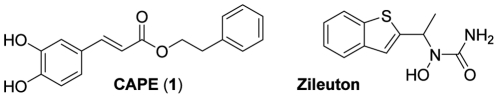
Molecular structures of CAPE 1 and zileuton.

A number of naturally-occurring compounds have been investigated as potential inhibitors of 5-LO and LT biosynthesis. Amongst these are polyhydroxylated products such as caffeic acid and related compounds that are widely distributed in plants and exhibit anti-oxidant [11]–[13] and anti-inflammatory properties [14], [15]. Synthetic caffeic acid analogues were recently shown to be promising 5-LO inhibitors [14], [16], [17], while caffeic acid and its naturally-occurring analogue, caffeic acid phenethyl ester (CAPE, Figure 1), a component of propolis from honeybee hives, were reported to inhibit LT production in mouse peritoneal macrophages [14].

Since many known 5-LO inhibitors, including zileuton [18], function by reducing the catalytically-active ferric form of 5-LO, we synthesized CAPE and some structural analogues to investigate their structure-activity relationship as free radical scavengers, antioxidants and 5-LO inhibitors. Both ester and amide analogues of CAPE were designed with the rationale that esters may be more susceptible to chemical and enzymatic degradation compared to the corresponding amide. Since the hydroxyl groups within the catechol moiety were reported to play an important role in several biological activities [19], cinnamoyl analogues were also synthesized to evaluate the effect of the presence of these functional groups.

In this study, our results demonstrate that while these compounds are effective antioxidants, certain structural features were required for effective inhibition of LT biosynthesis.

## Methods

### Ethics

Blood was obtained from health volunteer subjects after having obtained written consent. This research was approved by the «Comité d'éthique de la recherche avec les êtres humains» at Université de Moncton.

### Synthesis of CAPE-like analogues

**Figure 2:**
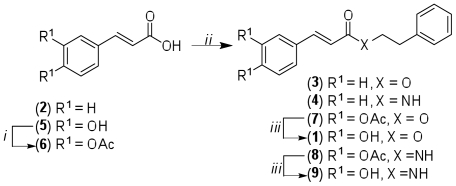
Summary of the synthesis of CAPE and its analogues.

The synthesis of CAPE and its analogues is summarized in Figure 2. The ester and amide analogues were synthesized from 2-phenylethanol or 2-phenylethanamine with cinnamic acid, **2**, or acetylated caffeic acid, **6**. The conversion of **2** or **6** into the corresponding carboxylic chloride was achieved by the Vilsmeier**-**Haack adduct [20] derived from thionyl chloride (SOCl_2_) and N,N-dimethylformamide (DMF) as catalyst. Base-induced de-*O*-acetylation in **7** or **8** to afford CAPE (**1**) and the amide analogue, **9**, was accomplished with potassium carbonate in methanol and dichloromethane (Figure 2). NMR and mass spectrometry analyses were found to be identical to those reported [21]–[24].

### Isolation of PMNL from peripheral blood

PMNL were isolated from heparinized blood obtained from healthy donors as previously described [25]. Briefly, blood was centrifuged at 300× *g* for 5 min at room temperature, plasma was collected and erythrocytes were removed by dextran sedimentation. Following centrifugation on a lymphocyte separation medium cushion (density, 1.077 g/ml) (Wisent, St-Bruno, Qc, Canada) at 900× *g* for 20 min at room temperature, PMNL (>96%) were obtained from the pellet after hypotonic lysis to remove residual erythrocytes.

### 5-LO activity in a cell-free assay

Investigation of compounds as 5-LO inhibitors was performed as described previously with minor modifications [16]. Briefly, HEK293 cells (ATCC, Manassas, VA) (10^7^ cells/ml) stably transfected with 5-LO were incubated in a hypotonic buffer (50 mM Tris-HCl pH 7.6, 150 mM NaCl, and 2 mM EDTA) for 10 min on ice. The cell mixture was then passed through a 21-gauge needle 10 times. Cell lysates were vortexed and centrifuged at 3800× *g* for 5 min at 4°C and the cell lysate supernatant containing 5-LO was recuperated. Supernatants containing 5-LO and 5 mM CaCl_2_ were then preincubated with each of the test compounds at the indicated concentrations (see figure legends) for 5 min at 37°C. The 5-LO reaction was initiated by the addition of 1 mM ATP and 40 µM AA followed by incubation at 37°C for 20 min. Reactions were stopped by the addition of 0.5 volume of cold MeOH∶CH_3_CN (1∶1) containing 50 ng of prostaglandin B_2_ (PGB_2_) as internal standard and samples were stored at −20°C overnight to maximize protein denaturation. Samples were then centrifuged at 1000× *g* for 10 min, the supernatant was diluted with 4 volumes of acidified water (acetic acid, 0.1% v/v) and then applied onto a preconditioned octadecyl (C_18_) column. Columns containing samples were washed with 2 ml acidified water and 5-LO products were eluted with 3 ml of methanol. After evaporation of solvents with nitrogen, products were suspended in 20% methanol and subjected to RP-HPLC analysis with diode array detection as previously described [26]. Total 5-LO products quantified represents the sum of LTB_4_, its trans isomers, 20-COOH- and 20-OH-LTB_4_ and 5-hydroxyeicosatetraenoic acid.

### Stimulation of PMNL for 5-LO products

Isolated PMNL (10^7^ cells/ml) suspended in Hank's balanced salt solution (Lonza, Walkerville, MD) were pre-incubated with compounds for 5 min at 37°C in the presence of 1.6 mM CaCl_2_ and 1 U/ml of adenosine deaminase (Sigma-Aldrich, Oakville, On, Canada). Cells were then stimulated for 15 min at 37°C with 1 µM thapsigargin (Sigma-Aldrich) with or without 10 µM AA (Cayman Chemical, Ann Arbour, MI) as previously described [27]. Reactions were stopped by the addition of 0.5 volume of cold MeOH∶CH_3_CN (1∶1) and 50 ng of PGB_2_ as internal standard and samples were stored at −20°C until processing on octadecyl (C_18_) columns and RP-HPLC analysis as indicated above.

### Measurement of AA release

Isolated PMNL (10^7^ cells/ml) were stimulated with 1 µM thapsigargin as above but in the presence of 0.1% of BSA to capture released AA and with a stimulation time of 5 min. Stimulation was stopped by the addition of 2 volumes of cold methanol, 300 ng octadeuterated-AA (Cayman Chemical) was added as an internal standard and samples were stored at −20°C overnight. Samples were centrifuged at 1000× *g* for 10 min and supernatants were diluted with 8 volumes of acidified water for processing on a preconditioned octadecyl (C_18_) as indicated above. Samples were eluted with 3 ml of methanol, dryed under N_2_, and pentafluorobenzylesters were prepared by adding 50 µl N,N-diisopropylethylamine (20% in CH_3_CN)(Sigma-Aldrich) and 50 µl 2,3,4,5,6-pentafluorobenzyl bromide (20% in CH_3_CN)(Sigma-Aldrich) [28]. After heating at 40°C for 40 min, samples were dried under N_2_, resuspended in 100 µl hexane and AA was measured by negative ion chemical ionisation gas chromatography/mass spectrometry using a TraceGC ultra column (Thermo, Waltham, MA) and a Polaris Q mass spectrometer (Thermo).

### *Ex vivo* whole blood stimulation

Zymosan stimulation of whole blood was performed as previously described [29], [30] with minor modifications. Each compound or its diluent dimethyl sulfoxide (DMSO) was added to 1 ml heparinized blood obtained from healthy donors at the indicated concentrations and incubated for 10 min at 37°C in a water bath. Blood was then stimulated with the addition of 125 µl of 40 mg/ml opsonised zymosan, gently vortexed and incubated for 30 min at 37°C. Samples were then centrifuged for 10 min at 960×g at 4°C. Plasma (350 µl) was removed and added to tubes containing 1.2 ml of CH_3_OH∶CH_3_CN (1∶1) and 50 ng of PGB_2_ as internal standard. Samples were stored overnight at −20°C and then processed for RP-HPLC analysis of 5-LO products as described above.

### Determination of the antioxidant and radical scavenging activity of test compounds

The antioxidant assay was performed as previously described [31]. Briefly, a 5 mM phosphate-buffered solution (pH 7.4) containing 0.05% Tween 20 (Sigma-Aldrich) and 0.16 mM linoleic acid (Cayman Chemical) was preheated at 40°C. Test compounds or their diluent (DMSO) were added to the mix at the indicated concentrations (see figure legends). The oxidation reaction, performed under a constant temperature of 37°C, was initiated by adding 50 µl of a 2,2′-azobis(2-amidinopropane) dihydrochloride (AAPH) solution (10 mg/ml) (Cayman Chemical) to 1 ml of the above solution. The rate of lipid oxidation was determined by measuring the absorbance at 234 nm with a Thermo Varioskan UV visible spectrophotometer every 5 min for 3 h. Inhibition of linoleic acid oxidation was calculated as followed: (%)  =  (1 - rate absorbance change with test compound/rate of absorbance change with solvent control) ×100.

The free radical scavenging activity of test compounds was measured as previously described using 2,2-Diphenyl-1-picrylhydrazyl (DPPH) as a stable radical [32], [33]. 1 ml of DPPH in ethanol (60 mM) was mixed with 1 ml of the test compounds at the indicated concentrations or their diluent (ethanol). Each mixture was then shaken vigorously and held in the dark for 30 min at room temperature. The absorbance of DPPH at 520 nm was then measured. The radical scavenging activity was expressed in terms of % inhibition of DPPH absorbance (%inhibition  =  [(A_control_−A_test_)/A_control_)]×100) where *A*_control_ is the absorbance of the control (DPPH solution without test compound) and *A*_test_ is the absorbance of the test sample (DPPH solution plus compound). Ascorbic acid was used as a reference compound.

### Statistical analysis

Statistical analysis and graph design were performed with GraphPad Prism 5 software (GraphPad Software, San Diego, California). All data are expressed as mean ± SEM. One sample t-tests were performed to determine significant difference from controls. IC_50_ values were calculated from a sigmoidal concentration-response curve-fitting model.

## Results

### The biosynthesis of 5-LO products in stimulated human PMNL

**Figure 3:**
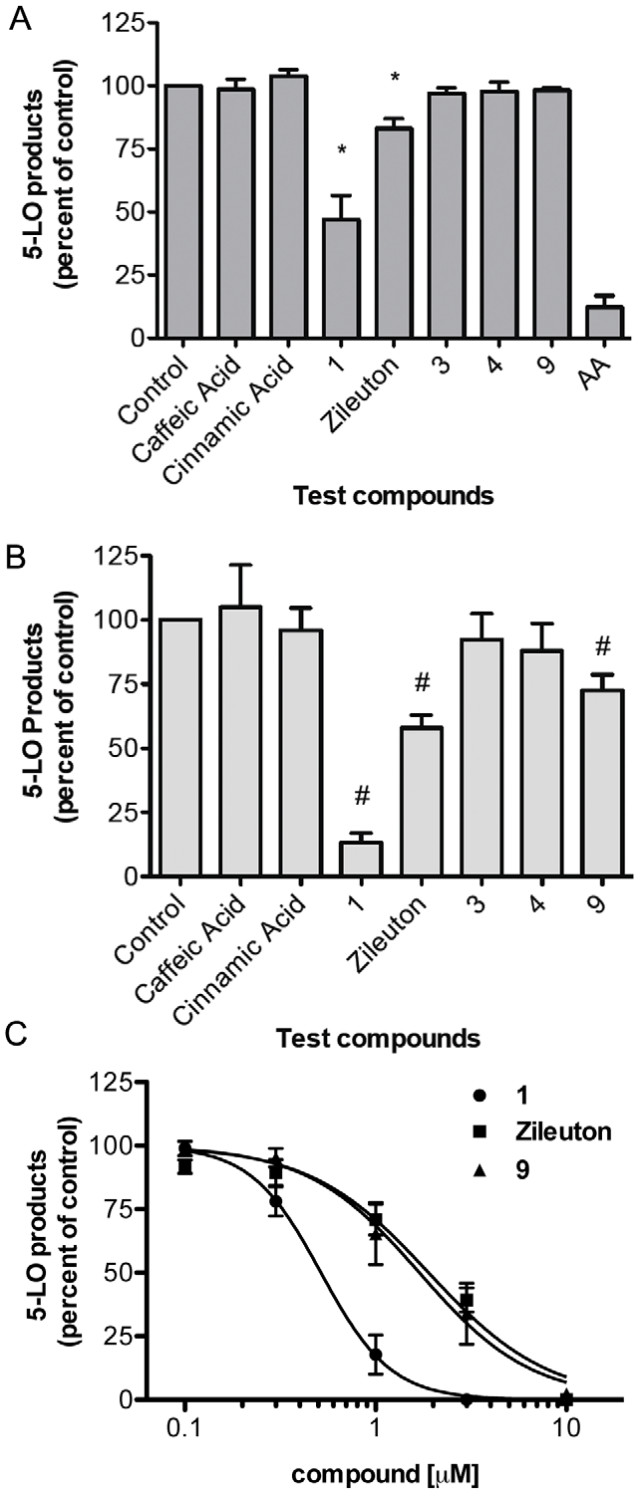
Biosynthesis of 5-LO products by thapsigargin-stimulated PMNL in the presence of various compounds. PMNL incubated with 1 µM of the indicated compounds or their diluent (Control, 0.5% DMSO) for 5 min were then stimulated with thapsigargin (1 µM) for 15 min in the presence (A) or absence (B) of exogenous arachidonic acid (10 µM). Dose-response of CAPE **1**, compound **9** and zileuton for the inhibition of the biosynthesis of 5-LO products (C). Reactions were stopped by the addition of 0.5 volume of cold MeOH∶CH_3_CN (1∶1) and samples were processed for measurement of 5-LO products by RP-HPLC. Total 5-LO products represent the sum of LTB_4_, its trans isomers, 20-COOH- and 20-OH-LTB_4_ and 5-hydroxyeicosatetraenoic acid. *Different from control, *P*<0.05, #different from control, *P*<0.005. AA = cells incubated without thapsigargin stimulus. Data are expressed as means ± SEM of 3 to 5 independent experiments, each performed in duplicate.

A first series of experiments was performed in which PMNL were stimulated with 1 µM thapsigargin in presence or absence of 10 µM AA. Under these conditions, the biosynthesis of 5-LO products was 176 ± 16 pmol/10^6^ cells (mean ± SEM) and 357±33 pmol/10^6^ cells (mean ± SEM) for thapsigargin and thapsigargin/AA, respectively. The effect of a fixed concentration (1 µM) of the various test compounds on the biosynthesis of 5-LO products was then measured (Figure 3). Stimulation of PMNL in the presence of exogenous AA excludes the possibility that the test compounds might affect LT biosynthesis by blocking AA availability. In PMNL stimulated with thapsigargin in the presence of exogenous AA, only CAPE **1** and zileuton significantly decreased production of 5-LO products by 53% and 17%, respectively (Figure 3A). A more significant decrease in the biosynthesis of 5-LO products was observed when PMNL were stimulated in the absence of exogenous AA. Under these experimental conditions, 1 µM of CAPE **1**, compound **9** and zileuton inhibited the biosynthesis of 5-LO products by 85%, 20% and 40%, respectively (Figure 3B). In concentration response experiments in the absence of exogenous AA, CAPE **1** showed potent inhibition of the biosynthesis of 5-LO products with an IC_50_ value of 0.52 µM while its amide analogue compound **9** and zileuton had IC_50_ values of 1.70 µM and 1.90 µM, respectively (Figure 3C and Table 1).

**Table 1:** IC_50_ values for the inhibition of the synthesis of 5-LO products of test compounds in the different assays.

Compounds		PMNL stimulation IC_50_ (µM)	Cell lysate IC_50_ (µM)	Whole blood IC_50_ (µM)
1	Mean	0.52	0.13	1.79
	CI	0.44 to 0.61	0.08 to 0.23	1.45 to 2.20
zileuton	Mean	1.90	3.54	1.41
	CI	1.48 to 2.42	2.34 to 5.38	1.22 to 1.63
9	Mean	1.70	2.38	4.93
	CI	1.21 to 2.38	1.43 to 3.95	3.42 to 7.10

Values are means from 3 independent experiments, each performed in duplicate.

CI = 95% confidence interval.

### 5-LO activity in a cell-free assay

**Figure 4:**
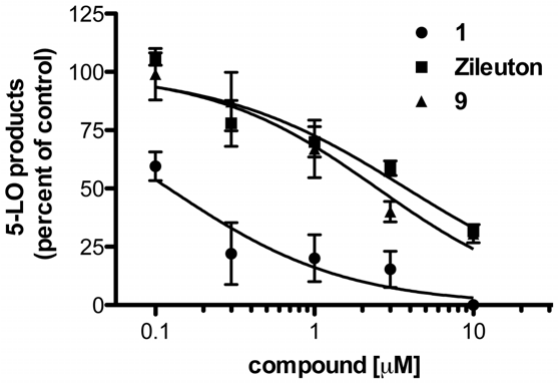
Impact of CAPE 1, compound 9 and zileuton on the synthesis of 5-LO products in cell lysates. HEK293 cell lysate supernatants were incubated with CAPE **1**, compound **9**, zileuton or their diluent (Control, 0.5% DMSO). Synthesis of 5-LO products was initiated by the addition of 40 µM AA and 1 mM ATP. Reactions were stopped after 20 min by the addition of 0.5 volume of cold MeOH∶CH_3_CN (1∶1) and samples were processed for measurement of 5-LO products by RP-HPLC. Total 5-LO products represent the sum of LTB_4_, its trans isomers, 20-COOH- and 20-OH-LTB_4_, and 5-hydroxyeicosatetraenoic acid. Values represent means ± SEM of three independent experiments, each performed in duplicate.

Since CAPE **1** and compound **9** inhibited LT biosynthesis in stimulated whole cells, their ability to inhibit 5-LO activity was investigated in a cell-free assay in HEK293 cells stably transfected with 5-LO. 5-LO activity was effectively inhibited in the presence of CAPE **1**, compound **9** and zileuton. CAPE **1** was a more potent 5-LO inhibitor than compound **9** and zileuton with a measured IC_50_ value that was an order of magnitude smaller (Figure 4 and Table 1).

### AA release from stimulated human PMNL

**Figure 5:**
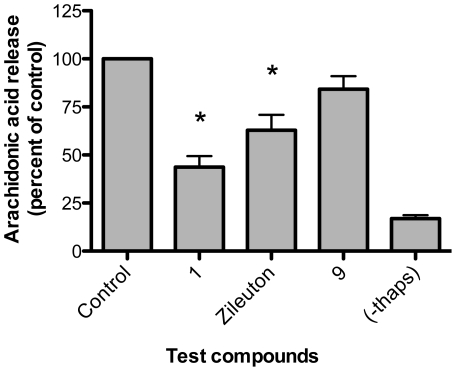
Impact of CAPE 1, compound 9 and zileuton on AA release by stimulated PMNL. PMNL were incubated with 1 µM of the indicated compounds or their diluent (Control, 0.5% DMSO) for 5 min and were then stimulated with thapsigargin (1 µM) or its diluent (-thaps) for 5 min. Stimulation was stopped by the addition of 2 volumes of cold methanol containing octadeuterated-AA as an internal standard. Samples were stored at -20°C overnight, AA was extracted on octadecyl columns, pentafluorobenzylesters were prepared and were measured by GC-MS. *Different from control, *P*<0.05. Data are expressed as means ± SEM of three independent experiments, each performed in duplicate.

The inhibition of LT biosynthesis by CAPE **1** and compound **9** was more effective in the absence of exogenous AA. We therefore investigated if these compounds might partially block LT biosynthesis by inhibiting the release of AA from membrane phospholipids, thus limiting substrate availability. Since zileuton has been shown to inhibit AA release from mouse peritoneal macrophages stimulated with zymosan [34], we investigated if our test compounds could also impact on this key cellular event in the biosynthesis of 5-LO products. When PMNL were pre-incubated with the test compounds (1 µM), only CAPE **1** and zileuton inhibited AA release by 56% and 37%, respectively, compared to controls (Figure 5).

### Biosynthesis of 5-LO products in stimulated whole blood

**Figure 6:**
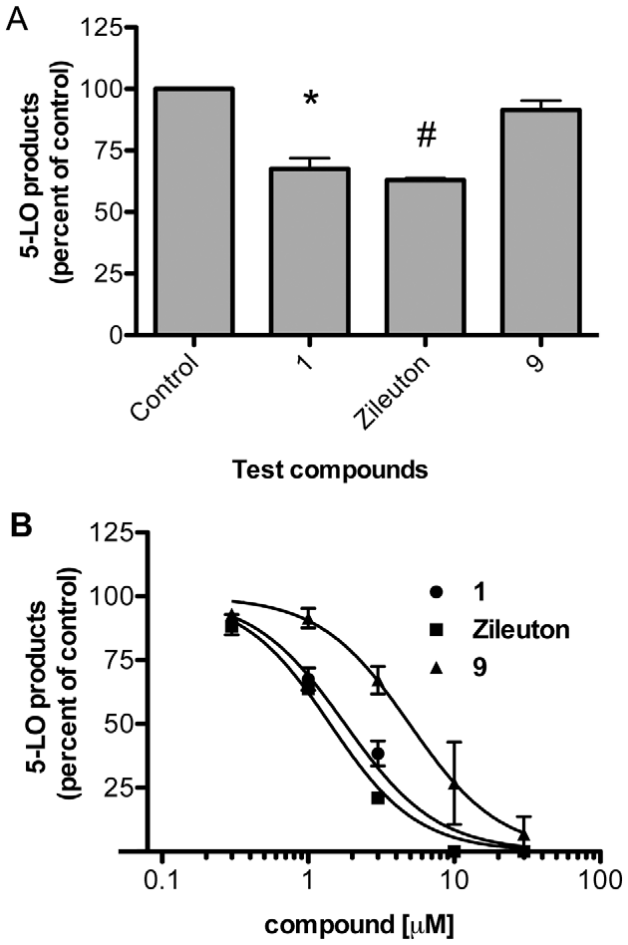
Impact of CAPE 1, compound 9 and zileuton on the biosynthesis of 5-LO products in stimulated whole blood. Whole blood incubated with 1 µM of the indicated compounds or their diluent (Control, 0.5% DMSO) for 5 min was then stimulated with opsonised zymosan (5 mg/ml) for 30 min (A). Dose-response for the inhibition of 5-LO products of test compounds in opsonised zymosan-stimulated whole blood (B). After stimulation, blood was centrifuged, plasma was removed and added to 3.5 volumes of cold MeOH∶CH_3_CN (1∶1) and samples were processed for measurement of 5-LO products by RP-HPLC. Total 5-LO products represent the sum of LTB_4_, its trans isomers, 20-COOH- and 20-OH-LTB_4_ and 5-hydroxyeicosatetraenoic acid. *Significantly different from control, *P*<0.05, #Significant different from control *P*<0.005. Data are expressed as means ± SEM of 3 independent experiments, each performed in duplicate.

To investigate the ability of the compounds that showed significant inhibition of LT biosynthesis in isolated PMN to inhibit LT biosynthesis in a more complex and physiologically-relevant environment, LT biosynthesis was measured *ex vivo* in stimulated human blood. When whole blood was stimulated with zymosan in the presence of 1 µM of the test compounds, CAPE **1** and the reference molecule zileuton had similar effects, inhibiting LT biosynthesis by 32% and 37%, respectively (Figure 6A). Dose-response experiments confirmed the similar capacity of 5-LO pathway inhibition by CAPE **1** (IC_50_ = 1.79 µM) and zileuton (IC_50_ = 1.41 µM) while compound **9** did inhibit 5-LO product biosynthesis at a higher concentrations (IC_50_ = 4.93 µM) (Figure 6B and Table 1). Importantly, in addition to thapsigargin stimulation of isolated PMNL, the use of opsonized zymosan in these whole blood experiments showed that the test compounds inhibit leukotriene biosynthesis in leukocytes activated by different stimuli.

### Antioxidant and free radical scavenging activity

**Figure 7:**
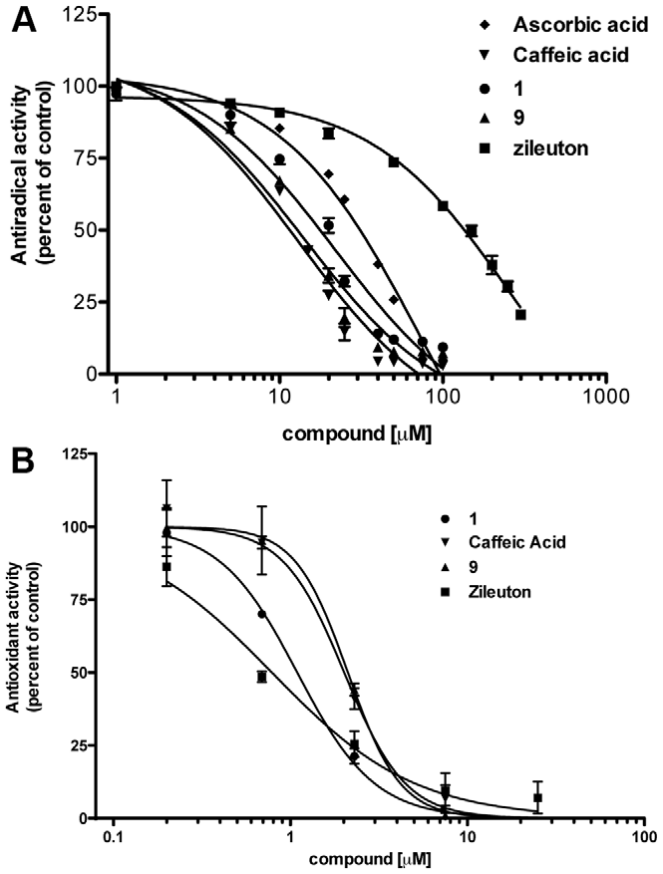
Free radical scavenging and antioxidant activities of various test compounds. (A) For free radical scavenging activity, 1 ml of DPPH (60 mM in ethanol) was mixed with 1 ml of the test compounds or their diluent (DMSO) in ethanol. Solutions were held in the dark for 30 min at room temperature and the absorbance was then measured at 520 nm. The free radical scavenging activity was expressed in terms of % inhibition of DPPH absorbance. (B) For antioxidant activity, test compounds or their diluent (DMSO) were added to a solution containing 0.16 mM linoleic acid and the oxidation reaction was initiated by adding 50 µl AAPH solution (10 mg/ml) to 1 ml of the above solution. The rate of lipid oxidation was determined by measuring the increase in absorbance at 234 nm over a 3 h period. Values represent the mean ± SEM of 3 independent experiments, each performed in triplicate.

One mechanism by which 5-LO can be inhibited is through reductive inhibition of the ferric non-heme iron of the enzyme. Compounds with anti-oxidant or free radical scavenging activity can therefore be effective 5-LO inhibitors. An initial evaluation of the reducing ability of the test compounds was determined by their interaction with the stable free radical DPPH since free radical scavengers can pair its free electron causing a stoichiometric decrease in absorbance at 520 nm. All catechol compounds tested, caffeic acid **5**, CAPE **1** and compound **9**, were efficient free radical scavengers in with IC_50_ values in the low µM range as opposed to their non-catechol analogues, cinnamic acid **2**, compounds **3** and **4** that showed no scavenging activity at concentrations up to 100 µM (Figure 7A and Table 2). Zileuton was not a strong free radical scavenger with an IC_50_ value of >100 µM while the reference compound ascorbic acid showed an IC_50_ values of 75 µM.

**Table 2:** IC_50_ values of test compounds as free radical scavengers and antioxidants.

		Free radical	Antioxidant
Compounds		scavenging	assay
		IC_50_ (µM)	IC_50_ (µM)
caffeic acid	Mean	13.2	2.01
	CI	9.76 to 17.9	1.68 to 2.42
cinnamic acid	Mean	n.i.*	n.i.**
	CI	n.i.*	n.i.**
1	Mean	21.9	1.09
	CI	15.2 to 31.4	0.77 to 1.54
zileuton	Mean	>100	0.79
	CI	n.i.*	0.58 to 1.06
3	Mean	n.i.*	n.i.**
	CI	n.i.*	n.i.**
4	Mean	n.i.*	n.i.**
	CI	n.i.*	n.i.**
9	Mean	14.01	2.11
	CI	9.95 to 19.7	1.76 to 2.54
ascorbic acid	Mean	75.4	n.t.
	CI	56.9 to 99.8	n.t.

Values are means from 3 independent experiments, each performed in duplicate.

CI = 95% confidence interval.

n.i. =  no inhibition at *100 µM or **25 µM.

n.t. =  not tested.

Since CAPE **1** and zileuton have documented antioxidant properties [13], [18], we investigated if CAPE **1** and its amide analogue compound **9** were better antioxidants than zileuton. As shown in Figure 7B and Table 2, CAPE **1**, compound **9**, Zileuton and caffeic acid all demonstrated similar antioxidant properties.

## Discussion

In our ongoing effort to develop more potent inhibitors of leukotriene biosynthesis, CAPE **1** and several of its analogues were synthesized and compared to the reference molecule zileuton. The present study focused on the inhibition of 5-LO and LT biosynthesis in human PMNL as these cells are important producers of the powerful chemoattractant LTB_4_ [35]. CAPE **1** was a more potent inhibitor than zileuton of 5-LO activity and of LT biosynthesis in stimulated human PMNL, although its inhibition of LT biosynthesis in whole blood was similar to that of zileuton. CAPE **1** had been previously reported to inhibit a plant lipoxygenase using linoleic acid as a substrate [13], [33]. Unfortunately, the authors reported this activity to be that of 5-LO. This was not the case since linoleic acid with its 9, 12 *cis* double bond is not a 5-LO substrate and the product of the plant lipoxygenase-catalyzed reaction, 9-hydroperoxy-10, 12-octadecadienoic acid, is not a 5-LO product. 5-LO catalyzes the abstraction of a pro-S hydrogen at the C-7 position of substrates with 5, 8 *cis* double bonds, like arachidonic acid, followed by the addition of molecular oxygen to form a 5-hydroperoxy-fatty acid [36]. Therefore, the present study is the first report of the inhibition of 5-LO activity by CAPE.

Several structural features of CAPE **1** were investigated for their importance in the inhibition of LT biosynthesis and of 5-LO activity. The phenethyl ester group of the molecule was essential for effective inhibition since caffeic acid and cinnamic acid did not show significant activity at concentrations up to 10 µM (data not shown), and as previously reported in 5-LO-transfected HEK293 cells [16]. This result in human leukocytes is not consistent with that reported in ionophore-stimulated murine peritoneal macrophages where both caffeic acid and CAPE show similar inhibition of leukotriene synthesis [14]; the reason for this difference is not apparent but the phenethyl moiety is clearly required for the inhibition of both 5-LO activity and LT biosynthesis in human cells. Similarly, the catechol moiety of the molecule appears to be essential for activity as compounds **3** and **4** were without inhibitory activity at concentrations up to 10 µM (not shown). While compound **9** inhibited LT biosynthesis and 5-LO activity, the presence of the amide linkage reduced its potency compared to the ester CAPE **1** by approximately 3-fold for LT biosynthesis and by 18-fold for the inhibition of 5-LO activity in cell lysates (Table 1).

Human PMNL were stimulated in the presence or in the absence of exogenous AA to evaluate the inhibition of LT biosynthesis while bypassing the critical step of AA release from phospholipids, or not. CAPE **1**, zileuton and compound **9** were all more effective inhibitors of LT biosynthesis in the absence of exogenous AA suggesting that all three compounds inhibit the 5-LO catalyzed conversion of AA to LTs. A cell-free 5-LO assay using HEK293 cells that were stably transfected with 5-LO confirmed that CAPE **1**, zileuton and compound **9** all inhibited the 5-LO-catalyzed conversion of AA to LTs, where CAPE **1** (IC_50_ = 0.13 µM) was 27-fold more active than zileuton for the inhibition of 5-LO activity while compound **9** and zileuton showed similar inhibitory activities.

It is well documented that human PMNL spontaneously release significant amounts of adenosine when they are cultured in vitro [37]. This build up is usually not observed in tissues and blood since stromal cells and erythrocytes rapidly transport adenosine into their cytosol [37], [38]. This adenosine acts through G-protein linked receptors to activate adenylate cyclase and increase cellular cAMP levels [27], [39]. In human PMNL, elevated cAMP reduces numerous functional responses to agonist stimulation including oxygen radical (superoxide) production, phagocytosis and leukotriene biosynthesis [27], [37], [38], [40]–[42]. Therefore, freshly isolated human PMNL will quite rapidly (within minutes) begin to lose their capacity to respond to agonists unless the accumulation of adenosine in cell culture is prevented. We routinely add ADA to isolated PMNL incubations to prevent the inhibitory constraint of adenosine [27], [42]–[47] and although the possibility exists that added ADA may interact with and impact on the test compounds in the present study, this is unlikely since the inhibition of 5-LO activity and LT biosynthesis were also measured in broken cell preparations and in stimulated whole blood, two assay conditions that were devoid of added ADA.

Since the 5-LO inhibitor zileuton was previously shown to also inhibit AA release from membrane phospholipids in mouse peritoneal macrophages [34], the release of free AA from PMNL phospholipids was also measured following cell stimulation. CAPE **1** (1 µM) inhibited almost 55% of the AA released from membrane phospholipids of stimulated human PMN compared to 35% of inhibition with zileuton and no effect of compound **9**. Since the group IVA phospholipase A_2_ (cPLA_2_α) is responsible for AA release in stimulated human PMNL [47], [48], these results suggest that both CAPE **1** and zileuton block LT biosynthesis in human PMN by inhibiting the activation of cPLA_2_α as well as that of 5-LO.

Many 5-LO inhibitors including zileuton [18] inhibit the enzyme by reducing the catalytically-active ferric form of 5-LO. Catechols as a class of compounds are known anti-oxidants and can potentially inhibit 5-LO as free radical scavengers and antioxidants. When the test compounds were evaluated for anti-oxidant and free-radical scavenging activity, their potency was not necessarily related to their ability to inhibit 5-LO. Not surprisingly, cinnamic acid and its phenethyl ester and phenethyl amide derivatives, compounds **3** and **4**, showed no antioxidant or free radical scavenging activity at concentrations up to 25 µM and 100 µM, respectively. However, caffeic acid, which showed no inhibition of 5-LO, was as effective an antioxidant and free-radical scavenger as CAPE **1** and compound **9**. This suggests that catechols may not efficiently reduce the ferric iron of 5-LO for enzyme inhibition without the contribution of a hydrophobic moiety that may act as an anchor to more specifically target the non-heme iron of the 5-LO protein. Zileuton was also an effective anti-oxidant as previously reported, but was not a good free radical scavenger.

The rationale for synthesizing amide-linked analogues was that they may be more stable than ester-linked compounds, like CAPE **1**, which could be susceptible to hydrolysis by esterases. Despite being much more potent than zileuton and compound **9** at inhibiting 5-LO activity in broken cell assays, and despite inhibiting the release of AA from stimulated cells, CAPE **1** was only moderately more effective than compound **9** at inhibiting LT biosynthesis in stimulated human PMNL and in whole blood, and was not different from zileuton in whole the blood assays. This suggests that while remaining a potent inhibitor of LT biosynthesis, the suspected susceptibility of CAPE **1** to esterases may reduce its potency in a physiological setting.

In summary, we characterized CAPE **1**, a naturally occurring component of propolis from honeybee hives, as a potent inhibitor of LT biosynthesis that acts as a dual inhibitor of 5-LO activity and of AA release from membrane phospholipids. A continued effort for the rational design of inhibitors of LT biosynthesis using the CAPE **1** structure as framework may yield stable and potent inhibitors of LT biosynthesis. Such rational design efforts will certainly be aided by the recent description of the crystal structure of the human 5-LO protein [49].
